# Avian Chaperonin Containing TCP1 Subunit 5 Supports Influenza A Virus Replication by Interacting With Viral Nucleoprotein, PB1, and PB2 Proteins

**DOI:** 10.3389/fmicb.2020.538355

**Published:** 2020-10-15

**Authors:** Xiaohan Zhang, Xian Lin, Chenghuang Qin, Kun Huang, Xiaomei Sun, Lianzhong Zhao, Meilin Jin

**Affiliations:** ^1^State Key Laboratory of Agricultural Microbiology, College of Veterinary Medicine, Huazhong Agricultural University, Wuhan, China; ^2^Key Laboratory of Preventive Veterinary Medicine in Hubei Province, The Cooperative Innovation Center for Sustainable Pig Production, Wuhan, China; ^3^Key Laboratory of Development of Veterinary Diagnostic Products, Ministry of Agriculture of the People’s Republic of China, Wuhan, China

**Keywords:** influenza viruses H5N6, nucleoprotein, polymerase basic protein 2, chaperonin containing TCP1 subunit 5, protein interaction

## Abstract

Humans and avian species are prone to influenza viral infection, which may cause serious clinical consequences. Many studies have documented the critical role of host factors in the influenza virus life cycle based on human models, but knowledge about their roles in birds is very limited. In this study, using immunoprecipitation coupled with mass spectrometry, a total of 72 potential interacting proteins of influenza nucleoprotein (NP) were identified in DF-1 cells. Among these proteins, avian chaperonin containing TCP1 subunit 5 (CCT5) was demonstrated to interact with influenza A virus (IAV) NP directly, as well as polymerase basic protein 1 (PB1) and polymerase basic protein 2 (PB2) but not with polymerase acidic protein (PA). Further investigation showed that viral infection profoundly elevated the expression level of cellular CCT5, whose expression, in turn, promoted the nuclear export of NP, as well as viral polymerase activity, thereby facilitating the replication of IAV. The obtained results suggested an important role of avian CCT5 in supporting influenza virus replication, which may serve as an anti-influenza target.

## Introduction

Influenza virus, a negative-sense RNA virus with a genome consisting of eight gene segments, is a highly dangerous pathogen circulated in both humans and animals around the world (Lamb and Choppin, 1983; Luo et al., 2018). To generate progeny virions, the influenza virus needs to hijack the host components to complete the synthesis of viral RNA (vRNA) and proteins (Herz et al., 1981; Jackson et al., 1982; O’Neill et al., 1995). Hence, a better understanding of how viral components co-opt and utilize cellular processes is critical for the elucidation of mechanisms of pathogenesis and for the development of novel antiviral drugs.

Viral RNP (vRNP) complex, which consists of a single-stranded negative-sense genomic RNA, multiple copies of the viral nucleoprotein (NP), and an RNA polymerase complex composed of polymerase basic protein 1 (PB1), polymerase basic protein 2 (PB2), and polymerase acidic protein (PA), is the basic unit of influenza A virus (IAV) transcription and replication (Jennings et al., 1983; Hsu et al., 1987; Klumpp et al., 1997). NP is an important multifunctional structural protein of the influenza virus that plays an important role in its life cycle (Liao et al., 2010). The NP mainly encapsulates the viral genome for RNA transcription, replication, and packaging (Ortega et al., 2000; Cros et al., 2005; Ozawa et al., 2007). In the past decade, many human factors have dynamically interacted with influenza components, as demonstrated through RNA interference (RNAi) screening, yeast two-hybrid analysis, and immunoprecipitation assay combined with liquid chromatography-tandem mass spectrometry (Stertz and Shaw, 2011; Roux et al., 2012; Stynen et al., 2012). Notably, the prevention or promotion of synthesized NP to the nucleus by host factors may greatly benefit or restrict viral replication (Görlich et al., 1995; Görlich and Kutay, 1999; Ozawa et al., 2007). However, studies on the involvement of avian host factors in interacting with influenza NP are limited.

In recent years, the H5N6 influenza virus has become highly pathogenic to birds and has dealt a major blow to the aquaculture industry, even causes death in humans (Bi et al., 2016). Birds, particularly waterfowl, play a central role in the preservation and transmission of the influenza virus (Hird et al., 2018). In this study, we investigated the interacting proteins of H5N6 IAV NP in chicken DF-1 cells by affinity purification-MS (AP-MS). Among these proteins, chaperonin containing TCP1 subunit 5 (CCT5), a member of the chaperone protein family, interacts with NP. Furthermore, we demonstrated that CCT5 expression is largely elevated by H5N6 viral infection, which reversely benefits its proliferation. Results present a new unrecognized avian host protein required for influenza viral replication in DF-1 cells.

## Materials and Methods

### Cells and Viruses

The chicken embryo fibroblast DF-1 cell lines and Madin-Darby canine kidney (MDCK) were purchased from the American Type Culture Collection (Manassas, VA, United States). All cells were grown in Dulbecco’s modified Eagle’s medium (DMEM)/high glucose (Hyclone™, USA) with 10% fetal bovine serum (Premium; PAN-Biotech, Germany) and maintained at 37°C in 5% CO_2_. The IAVs used were A/duck/Hubei/WH18/2015 (H5N6), A/duck/Hubei/Hangmei01/2006 (H5N1-HM), and A/chicken/Hubei/327/2004 (H5N1-DW). Green fluorescent protein (GFP) recombinant H5N6 virus (H5N6-GFP) was constructed in this study, according to a previous method (Perez et al., 2013). All viruses were amplified using 10-day-old embryonic chicken eggs and stored in our laboratory.

### Plasmids and Small Interfering RNAs

The RNA of DF-1 cells was extracted and converted to complementary DNA (cDNA) by 18 T primers. The full-length CCT5 gene was amplified by PCR using the cDNA as a template and inserted into p3XFlag- and pCAGGS-HA-expressing vector. At the same time, H5N6 virus NP and PB2 coding sequences were cloned into the pCAGGS-HA vector for overexpression. The cloning primers are provided in extended materials, and all constructs were verified by Sanger sequencing. pPol I-AVI plasmid was kindly provided by Hualan Chen (Harbin Veterinary Research Institute, Harbin, China).

All designed small interfering RNAs (siRNAs) or negative control RNA (NC) used in this study were synthesized by GenePharma (Shanghai, China). The PCR primers and siRNA sequences are listed in the Supplementary Material.

### Transfection

All plasmids and siRNAs used were transfected using Lipo8000™ transfection reagent (C0533, Beyotime, China) following the manufacturer’s guides.

### Protein Coimmunoprecipitation and Mass Spectrometry

To obtain NP-associated host factors, we performed immunoprecipitation-mass spectrometry (IP/MS) experiments. DF-1 cells were seeded into six-well plates with an initial concentration of 1 × 10^5^ cells/well and transfected with hemagglutinin-nucleoprotein (HA-NP) or pCAGGS-HA empty vectors with 2 μg per well by using Lipo8000™ transfection reagent for 24 h. DF-1 cells were subsequently subjected to phosphate-buffered saline (PBS) wash and radioimmunoprecipitation assay (RIPA) lysis buffer (p0013, Beyotime, China). They were then incubated with 20 μl HA-labeled agarose beads (sc-500777, Santa Cruz) for 2 h and rotated at 4°C. The agarose beads were washed five times with buffer cells lysis buffer and eluted by centrifugation. The samples were boiled with sodium dodecyl sulfate-polyacrylamide gel electrophoresis (SDS-PAGE) sample loading buffer and subjected to SDS-PAGE analysis for Western imprinting and silver staining assay. Differential bands in HA immunoprecipitates (HA-IP) lanes were cut and sent for tryptic digestion. After reduction and alkylation, trypsin (mass ratio, 1:50) was added and hydrolyzed at 37°C for 20 h. After desalination, the enzymatic hydrolysis product was lyophilized and redissolved in 0.1% formic acid solution. The tandem mass spectrometry (MS/MS) signals were processed against the Uniprot Gallus protein database (36,624) using the Mascot 2.2 algorithm with the following parameters: variable modifications, oxidation (Met), N-acetylation, pyroglutamination (Gln); maximum missed cleavages, 2; peptide mass tolerance, 100 ppm; and MS/MS tolerance, 0.5 Da. Protein identification was based on the criterion of having at least one MS/MS data signal with Mascot scores that exceeded the thresholds (*p* < 0.05).

### Western Blot Analysis

To evaluate the expression of genes at the protein level of samples in this study, we extracted the proteins by using cell lysis buffer with protease inhibitor cocktail (B14001, Biomake, United States), boiled them with sample loading buffer, separated by 10% SDS-PAGE, and transferred them into nitrocellulose membranes (10,600,001, GE Whatman, Germany). After blocking with 2% bovine serum albumin (BSA, United States), the membranes were incubated with antibodies against glyceraldehyde-3-phosphate dehydrogenase (GAPDH), CCT5, phenylalanyl-tRNA synthetase, alpha subunit (FARSA), RuvB-like AAA ATPase 1 (RUVBL1), NOP56, methylenetetrahydrofolate dehydrogenase (NADP+ dependent) 1-like (MTHFD1L), influenza NP, influenza PB2, influenza PA, influenza PB1, or LaminiB1. After washing with PBST, the membranes were incubated in horseradish peroxidase (HRP)-conjugated secondary antibody. Finally, the signals were detected using an Immobilon Western Chemiluminescent HRP substrate kit (Thermo Fisher) and imaged by ChemBis (Eastwin). The antibodies specific for influenza NP, PA, PB2, and PB1 proteins were purchased from GeneTex (Irvine, CA, United States), and anti-CCT5 and anti-LaminiB1 antibodies were obtained from Proteintech (United States). The relative integrated optical density of the blot images was quantified using Image-Pro Plus software, as shown below each blot.

### Real-Time Quantitative PCR Assays

Cell total RNA was isolated using the TRIzol reagent according to the manufacturer’s instructions. Exactly 2 μg of total RNA was used to generate cDNA by avian myeloblastosis virus reverse transcriptase (AMV; TaKaRa Biotechnology, Dalian, China) and an oligo(dT)18 primer. Viral RNA and complementary RNA (cRNA) of the influenza virus were reverse transcribed using corresponding primers as listed in the extended material. Real-time quantitative PCR (qRT-PCR) was performed on an ABI ViiA7 Instrument (Applied Biosystems) using SYBR green detection chemistry (Roche). Relative expression levels were calculated by applying the 2^−ΔΔCt^ method using the reference gene relative to the control samples.

### Viral Growth Curve and Flow Cytometry Analysis

siCCT5#1 or NC was delivered into six-well plates, and DF-1 cells were cultured at a concentration of 60 nM per well by using Lipo8000™ transfection reagent. At 24 h post-transfection, the cells were infected with the H5N6 influenza virus with 0.01 multiplicity of infection (MOI). Cell culture medium was collected at 12, 24, and 36 h after infection, and virus titer was determined by plaque assay on MDCK cells. DF-1 cells were transfected with siCCT5 or NC for 24 h. After that, the cells were digested with 0.25% trypsin and 0.02% ethylenediaminetetraacetic acid (EDTA; GENOM, China) and fixed with 4% paraformaldehyde (Biosharp, China). The fluorescence intensity was measured by flow cytometry (BD FACSVerse™ Flow Cytometer, United States).

### Cell Viability Assay

Cell counting kit 8 (CCK-8, Donjindo) was utilized to examine the cell viability of siCCT5#1 transfected DF-1 cells according to the manufacturer’s protocols. DF-1 cells were seeded into 96-well plates at an initial concentration of 1 × 10^3^ cells/well. After incubation for 12, 24, and 36 h, CCK-8 solution was added to each well, and cells were incubated further for 1 h. The absorbance at 450 nm was measured using SPARK 10 M (Tecan Austria GmbH Untersbergstr, Austria).

### Immunofluorescence Assay

The cells were fixed with 4% paraformaldehyde and permeabilized with 0.2% Triton-X100. Then, the cells were incubated with PBS containing 1% BSA at room temperature for 1 h. Subsequently, cells were incubated with indicated primary antibody at room temperature for 2 h and with the fluorescent-labeled second antibody for another 2 h at room temperature. The nuclei were stained with 4’,6-diamidino-2-phenylindole (DAPI) reagent. Images were obtained under the laser confocal microscope (LSM510 or LSM880; Zeiss, Germany). The fluorescent-labeled second antibodies used in this study were Alexa Fluor 488-conjugated AffiniPure goat antirabbit, Alexa Fluor 488-conjugated AffiniPure goat antirabbit, and CoraLite594-conjugated goat antimouse IgG (SA00006-2 and SA00013-3; Proteintech, United States).

### Nuclear and Cytoplasmic Fractionation

DF-1 cells (10^6^) were collected at 3, 6, and 9 h postvirus infections and subjected to nuclear and cytoplasmic fractionation. Briefly, the harvested cells were lysed on ice for 30 min using 50 μl of cytoplasmic extraction buffer (Invent Biotechnologies, Inc. United States, SC-003) containing proteinase inhibitors cocktail. The cell suspension was vortexed and centrifuged for 10 min at 3,500 *g* at 4°C. The supernatants were then collected as the cytoplasmic fraction. The nuclear pellet was washed three times with cold PBS and then dissolved using 50 μl cell lysis buffer (Beyotime, p0013) as the nuclear fraction.

### Viral Polymerase Activity Assay

pPol I-AVI (0.6 μg) together with 0.6 μg Flag-CCT5 or empty vector was cotransfected into 12-well plated DF1 cells using Lipofectamine 8000. After transfection for 12 h, cells were infected with 0.1 MOI H5N6-WT virus. The samples were harvested and lysed at 12 h after infection, and the luciferase activity was measured using the Dual-Luciferase Reporter Assay Kit (Promega E1910 and E1960). The protein levels of CCT5 and viral NP, PA, PB1, and PB2 were determined by Western blotting; GAPDH was used as a loading control.

### Statistics

Significance value was calculated in GraphPad Prism software (San Diego, CA, United States) using the Student’s *t*-tests. Data were shown as means ± SD collected from three independent experiments. *p* < 0.05 was considered statistically significant.

## Results

### Identification of Host Proteins Interacting With the NP of Influenza A Virus in DF-1 Cells

**Figure 1:**
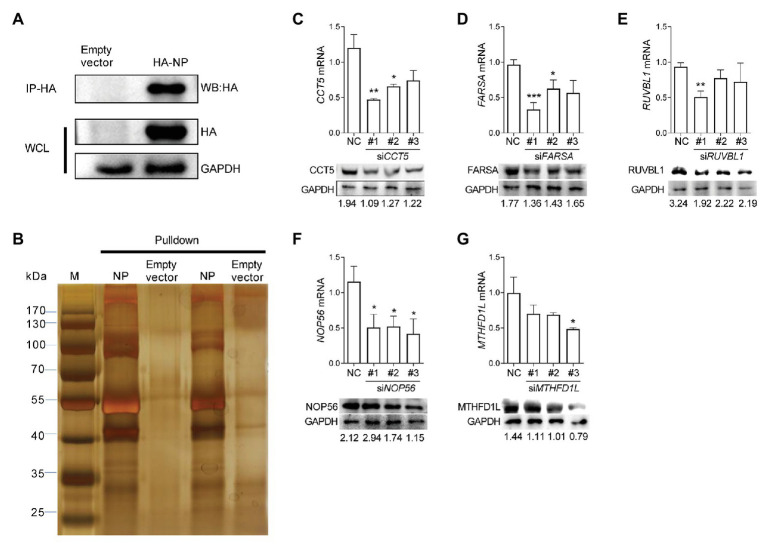
Identification of influenza virus nucleoprotein (NP)-associated factors by immunoprecipitation-mass spectrometry (IP/MS). **(A,B)** Eluates of duplicate hemagglutinin (HA) immunoprecipitates from HA-NP overexpressed DF-1 cells were analyzed by sodium dodecyl sulfate-polyacrylamide gel electrophoresis (SDS-PAGE) and silver staining. **(C–G)** DF-1 cells were transfected with three different chaperonin containing TCP1 subunit 5 (CCT5), phenylalanyl-tRNA synthetase, alpha subunit (FARSA), RuvB-like AAA ATPase 1 (RUVBL1), NOP56, methylenetetrahydrofolate dehydrogenase (NADP+ dependent) 1-like (MTHFD1L) small interfering RNAs (siRNAs) as indicated for 24 h. Knockdown efficiency was determined by real-time PCR and Western blot. The data are presented as the means ± SD from three independent experiments (^*^p < 0.05; ^**^p < 0.01; ^***^p < 0.001, by *t*-test).

The understanding of interactions between IAV components and its specific avian host partner could provide new insights into the mechanisms by which viruses promote their intracellular life cycle. To explore the potential host proteins that interact with IAV nucleoprotein NP, we performed IP assay in DF-1 cells transfected with a plasmid expressing HA-labeled H5N6 influenza NP (HA-NP). The transfection of the pCAGGS-HA empty vector was set as a negative control. After that, the HA-NP and its interacting components were enriched and eluted using anti-HA agarose beads. The obtained protein mixture was further subjected to Western blot and silver staining analyses (Figures 1A,B). Several specific bands were observed in the HA-NP group compared with the control group, which were incised and analyzed by liquid chromatography-tandem MS (LC-MS/MS). A total of 72 host proteins were identified (Supplementary Table 1). To narrow down the potential candidate quantities, we overlapped the identified proteins with the published influenza-host interactome results by Heaton et al. (2016). Eventually, CCT5, FARSA, MTHFD1L, NOP56, and RUVBL1 ranked high scores and were chosen for further function verification (Supplementary Table 2).

**Figure 2:**
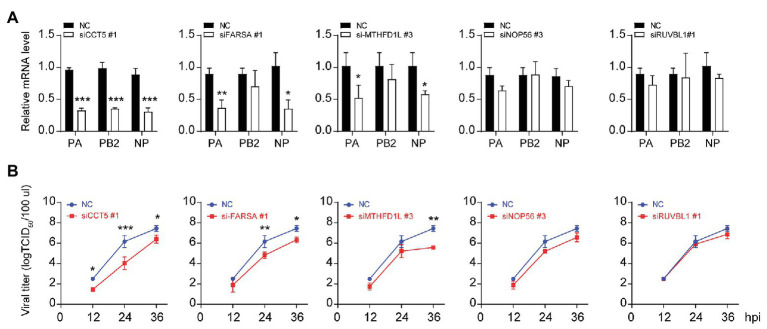
The loss of CCT5 restricts H5N6 replication in DF-1 cells. **(A,B)** Viral nucleoprotein (NP)/polymerase acidic protein (PA)/polymerase basic protein 2 (PB2) messenger RNA (mRNA) abundance in DF-1 cells that were transfected with indicated siRNAs or a negative control followed by infection with 0.1 multiplicity of infection (MOI) H5N6-GFP virus. The cell supernatants were collected at 12, 24, and 36 h postinfection and subjected to TCID_50_ measurement in Madin-Darby canine kidney (MDCK) cells (^*^p < 0.05; ^**^p < 0.01; ^***^p < 0.001, by *t*-test).

Three different siRNAs targeting each candidate genes were designed to knock down their expression in DF-1 cells; however, the most efficient one was selected for further experiments (Figures 1C–G). Using these siRNAs, we investigated the effect of reduced CCT5, FARSA, MTHFD1L, NOP56, and RUVBL1 expression on viral NP, PA, and PB2 messenger RNA (mRNA) *via* qRT-PCR. Among these tested genes, knockdown of CCT5, FARSA or MTHFD1L, but not NOP56 or RUVBL1, could cause a considerable decrease in NP, PA, or PB2 mRNA abundance compared with the NC control, which indicates their close relationships with viral replication (Figure 2A). Simultaneously, the supernatants’ viral titers were also calculated by the determination of TCID_50_, the results of which were consistent with mRNA expression levels (Figure 2B). Moreover, knockdown of the expression of CCT5 resulted in a decrease in fluorescence intensity, which reflected the reduced H5N6-GFP viral load (Supplementary Figure 1). Taken together, the involvement of CCT5 in influenza infection might be an important event that warrants our further study.

### CCT5 Interacts With Viral NP, PB1, and PB2 But Not With PA

**Figure 3:**
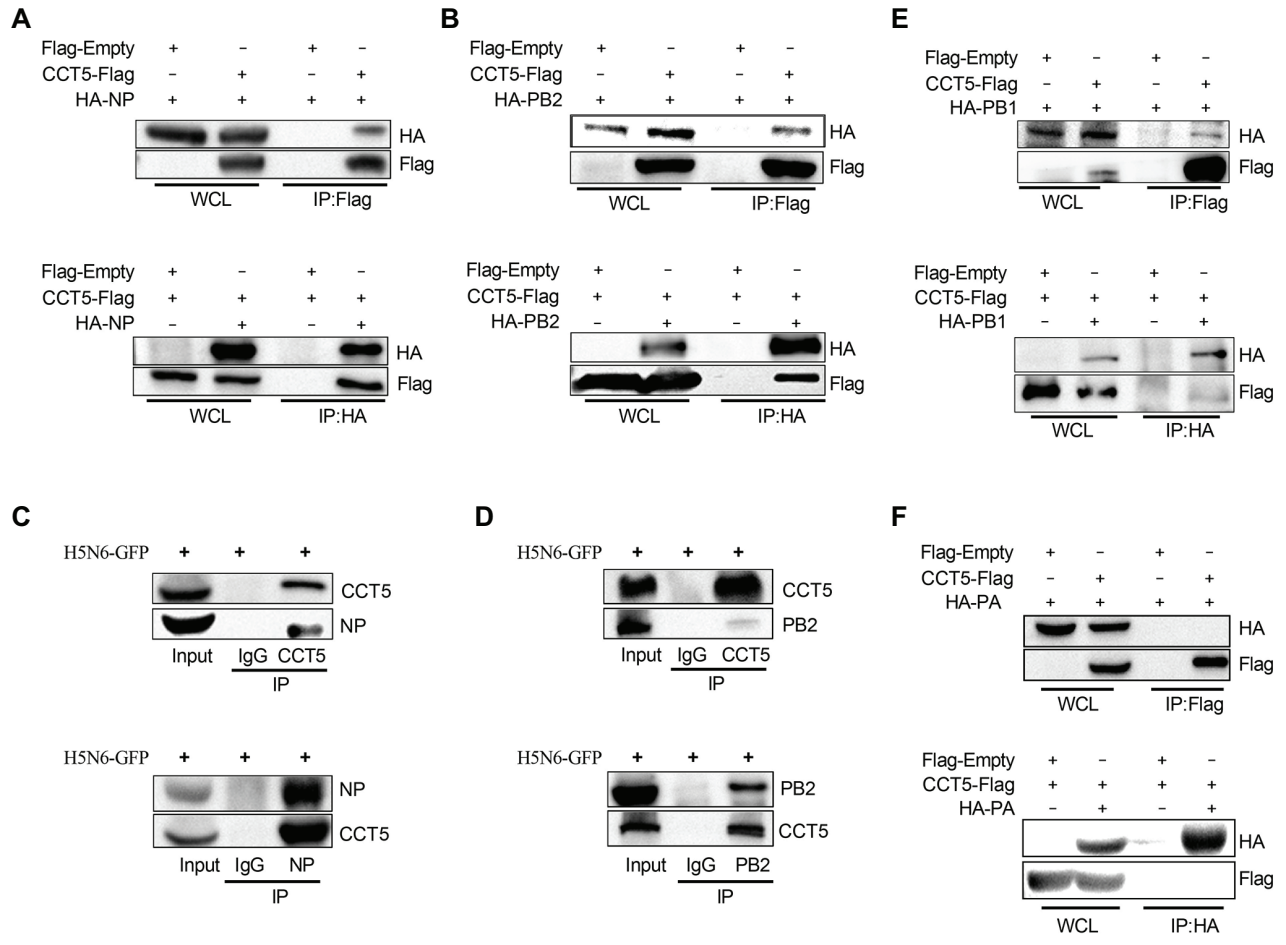
Chaperonin containing TCP1 subunit 5 is associated with viral nucleoprotein (NP), polymerase basic protein 2 (PB2), and polymerase basic protein 1 (PB1) but not polymerase acidic protein (PA) by coimmunoprecipitation (co-IP) assay. **(A,B)** Hemagglutinin NP (HA-NP) or HA-PB2, together with CCT5-Flag, were transfected into DF-1 cells for 24 h. Sample lysates were incubated using agarose beads pretreated by HA or Flag antibodies for 2 h at room temperature. The IP eluates were subjected to Western blot analysis. **(C–F)** DF-1 cells were infected with 0.1 MOI H5N6-GFP for 12 h. Co-IP experiment was performed as described in the Materials and Methods section, and the IP products were subjected to Western blot analysis. Immunoglobulin G (IgG) antibody coprecipitation was used as the control group. The presented results were from three independent experiments.

Next, we examined whether CCT5 is associated with viral NP. First, HA-NP or HA-PB2 and Flag-tagged CCT5 were cotransfected in DF-1 cells followed by coimmunoprecipitation assay using anti-Flag or anti-HA tag monoclonal antibodies, in which empty vectors were used as controls. The results showed that HA-NP could be precipitated with anti-Flag antibody upon cotransfection with Flag-CCT5 but not with the empty vector. A similar result was observed in Flag-CCT5, precipitated by the anti-HA antibody in the presence of HA-NP (Figure 3A). The study of Fislova et al. (2010) had indicated that PB2 proteins from various influenza virus subtypes could associate with CCT protein. In line with this conclusion, the PB2 protein of the H5N6 virus could be pulled down by CCT5 and vice versa (Figure 3B). Supportively, in H5N6-infected DF-1 cells, endogenous CCT5 is also shown to interact with viral NP and PB2 proteins (Figures 3C,D). Furthermore, the interaction between CCT5 and the other two components of the RNP was further tested in H5N6-infected DF-1 cells. Interestingly, PB1 but not PA could be pulled down by endogenous CCT5 (Figures 3E,F). These results suggested that CCT5 was closely involved in the influenza life cycle in DF-1 cells.

**Figure 4:**
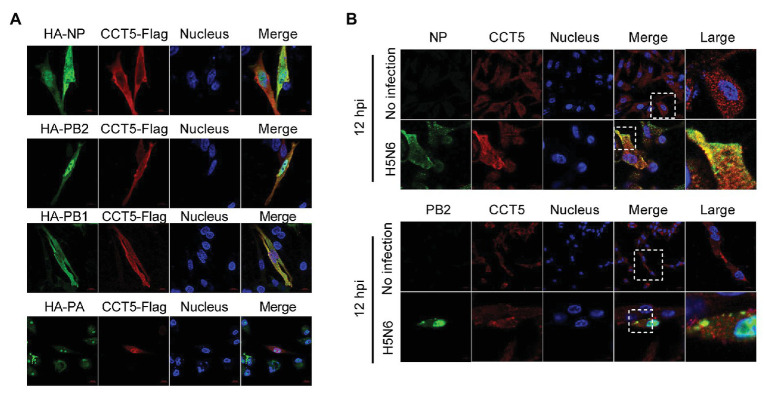
Colocalization of nucleoprotein (NP), polymerase basic protein 2 (PB2), and polymerase basic protein 1 (PB1) with CCT5 protein. **(A)** CCT5-Flag and HA-NP, HA-PB2, HA-PB1, or HA-PA were cotransfected into DF-1 cells for 24 h. CCT5-Flag and HA-NP, HA-PB2, HA-PB1, or HA-PA were labeled with anti-Flag mouse and anti-HA rabbit antibodies and stained with antimouse 594 red fluorescent secondary antibody and 488 green fluorescent antirabbit antibodies, respectively. The nucleus was stained with 4’,6-diamidino-2-phenylindole (DAPI). The red, green, blue, and yellow fluorescence represents the CCT5-Flag, the HA-NP, HA-PB2, HA-PB1, or HA-PA, the localization of the nucleus, and the colocalization region, respectively. **(B)** DF-1 cells were infected with or without the H5N6-WT virus, and then, anti-CCT5 rabbit red fluorescent antibody and anti-NP/PB2 mouse green fluorescent antibody were used for immunostaining. The white boxed region was enlarged, as shown in the right. These experiments were examined with a confocal microscope (LSM 880; Zeiss) from three independent experiments.

Given the interaction between CCT5 and the viral proteins, the colocalization between the interacting partners should be observed in immunofluorescent staining. To verify their interaction, HA-NP, HA-PB2, HA-PB1, or HA-PA combination with Flag-CCT5 plasmids were cotransfected into DF-1 cells. The data showed that CCT5 was located in both cytoplasm and nucleus. More importantly, in line with coimmunoprecipitation (co-IP) results, CCT5 could colocalize with viral NP, PB2, and PB1 but not with PA in transfected cells (Figure 4A). The colocalization of endogenous CCT5 with NP or PB2 was further confirmed in the H5N6 virus-infected DF-1 cells (Figure 4B). The above results demonstrated that CCT5 interacts with viral NP, and PB1 and PB2, which suggested a potential role of CCT5 in response to influenza virus infection.

### IAV Infection Upregulates CCT5 Expression in DF-1 Cells

**Figure 5:**
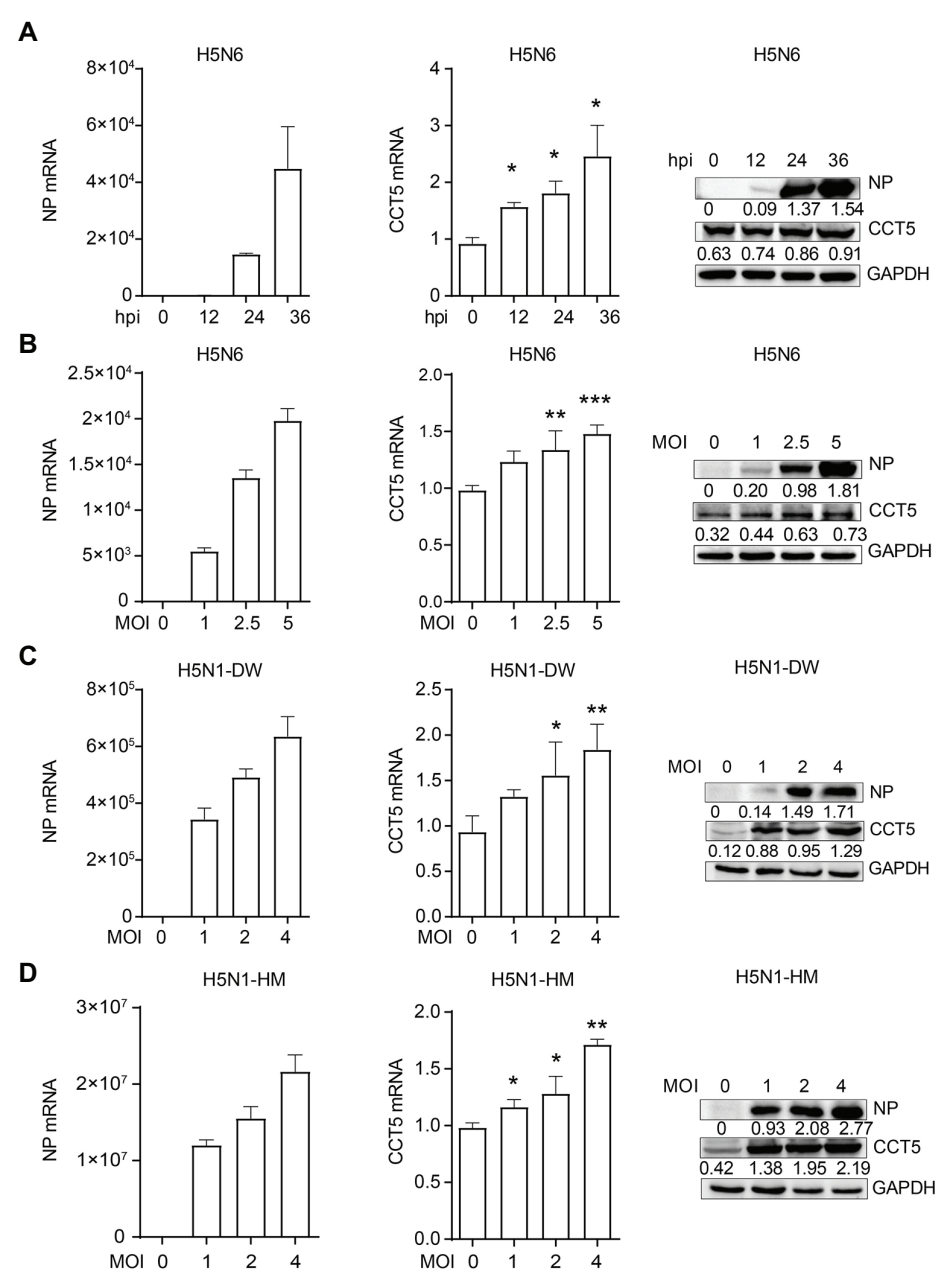
Influenza virus infection promotes the expression of CCT5 in DF-1 cells. **(A)** DF-1 cells were infected with or without 0.1 MOI H5N6-GFP virus for 12, 24, and 36 h, respectively. The mRNA and protein levels of CCT5 and nucleoprotein (NP) were measured by real-time PCR and Western blot analysis. **(B–D)** DF-1 cells were infected with the indicated dose of H5N6-GFP, H5N1-HM, or H5N1-DW virus for 12 h; the mRNA and protein levels of CCT5 and NP were detected by real-time PCR and Western blot, respectively. Glyceraldehyde-3-phosphate dehydrogenase (GAPDH) was used as a loading control for protein. Chicken ACTIN gene was used to normalize mRNA expression (^*^p < 0.05; ^**^p < 0.01; ^***^p < 0.001; from three independent experiments by two-tailed Student’s *t*-test).

Next, we wondered whether the expression of CCT5 was regulated during the influenza virus. To verify this hypothesis, DF-1 cells were infected with the H5N6 virus, and then, the mRNA and protein levels of both CCT5 and NP were analyzed. The abundance of CCT5 mRNA and protein were elevated in H5N6-infected DF-1 cells, and this parameter is positively correlated with viral replication during the infection course (Figure 5A). Moreover, higher initial H5N6 virus challenge gave rise to a more increased cellular CCT5 expression at both mRNA and protein levels (Figure 5B). Next, to determine whether the upregulation of CCT5 was specific to H5N6, we selected two additional IAV strains, H5N1-HM and H5N1-DW, for further evaluation. Similar to H5N6, either H5N1-DW or H5N1-HM infection was able to result in an increased expression of CCT5 at both mRNA and protein levels (Figures 5C,D). These results indicated that the expression of CCT5 was upregulated in avian host cells exposed to IAV infection.

### The Knockdown of CCT5 Restricts IAV Replication in DF-1 Cells

**Figure 6:**
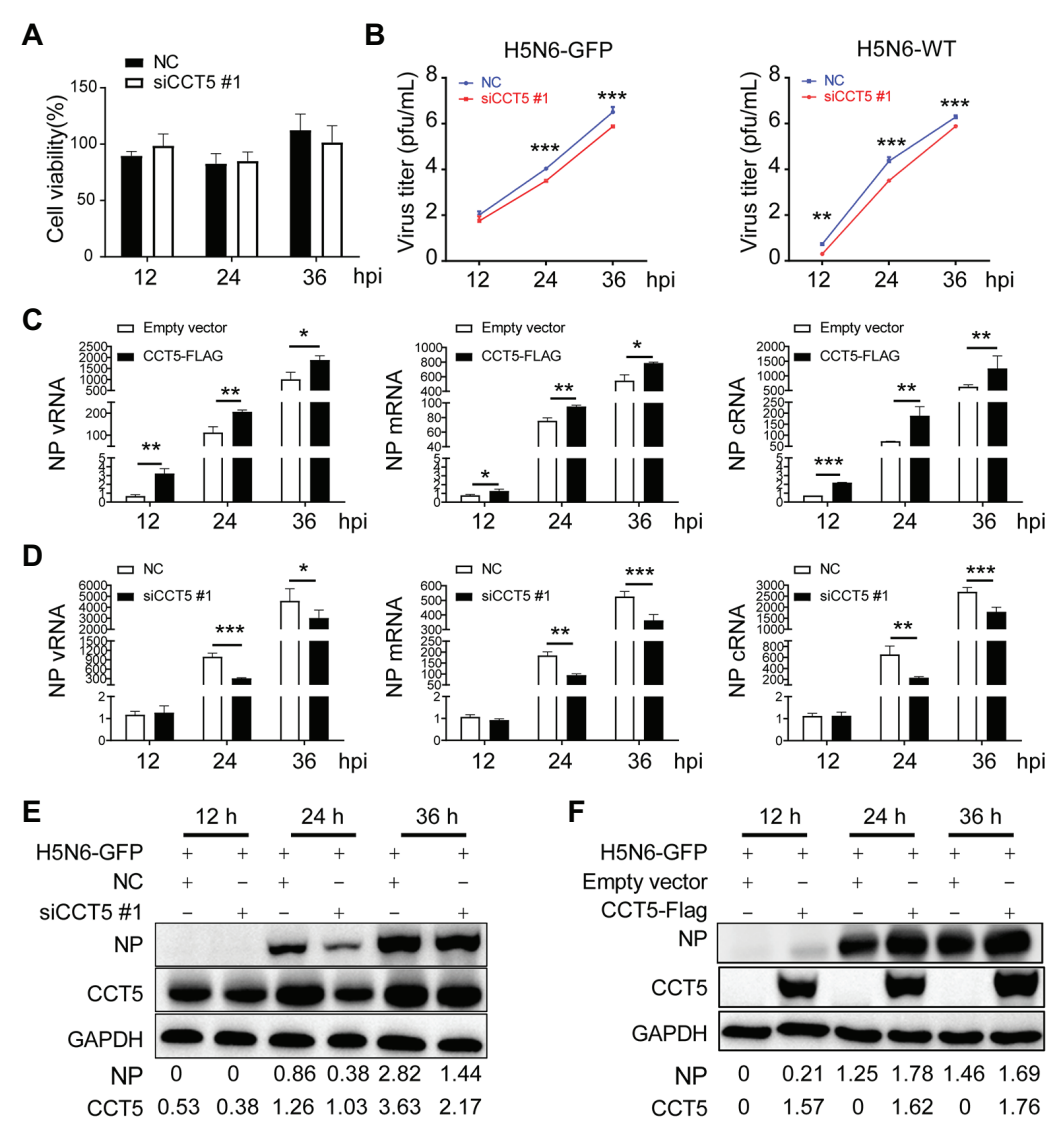
Silencing CCT5 inhibits influenza virus replication in DF-1 cells. **(A)** DF-1 cells were transfected with siCCT5#1 or NC for 12 h, and the cell viability was measured by CCK-8. **(B)** Growth curves of H5N6-WT and H5N6-GFP virus were determined in CCT5 knockdown DF-1 cells. **(C,D)** Effect of overexpressed or suppressed CCT5 on viral RNA (vRNA) synthesis. DF-1 cells transfected with CCT5-Flag or siCCT5#1 followed by infection with 0.1 MOI H5N6-GFP virus. The levels of nucleoprotein (NP) vRNA, complementary RNA (cRNA), and mRNA were determined by real-time PCR. **(E,F)** NP protein levels were also monitored by Western blot analysis. GAPDH was used as a loading control and marker. GAPDH was used as a loading control for protein. Chicken ACTIN gene was used to normalize mRNA expression (^*^p < 0.05; ^**^p < 0.01; ^***^p < 0.001; from three independent experiments by *t*-tests).

Considering that H5N6 promoted CCT5 expression, we focused our concentration on determining the potential effect of CCT5 on influenza virus replication. First, to rule out the possibility that silencing CCT5 may cause poor viability of DF-1 cells, we carried out cell viability assay. CCT5 depletion had no obvious influence on cell viability (Figure 6A). We further challenged siCCT5 or NC-transfected DF-1 cells with H5N6-GFP or H5N6-WT virus at an initial MOI of 0.1 for 12, 24, and 36 h. Then, we harvested the culture supernatants for viral titer measurement at indicated time points and measured the fluorescence intensity of the infected cells collected at 24 h postinfection; the uninfected DF-1 cells were used as a negative control. In the H5N6 infection group, the cell pellet was collected for Western blot and qPCR analysis at indicated time points. The data showed that the impaired expression of CCT5 significantly decreased viral titers in culture supernatants after 24 and 36 h compared with NC transfection (Figure 6B). Besides, the reduced expression of CCT5 resulted in a decreased abundance of viral NP vRNA, mRNA, cRNA, as well as NP protein (Figures 6D,E).

Conversely, ectopically expressed CCT5 profoundly enhanced H5N6 replication by increasing vRNA and protein levels (Figures 6C,F). To determine if CCT5 has broad effects on influenza virus replication, we additionally evaluated knockdown of CCT5 on the proliferation of another two subtypes of the influenza virus, H5N1-HM and H5N1-DW, in DF-1 cells. The results demonstrated that the depletion of CCT5 significantly attenuated the growth of both viruses (Supplementary Figure 2). Overall, the data suggested that CCT5 played an important role in supporting IAV replication in DF-1 cells.

### CCT5 Promotes NP Nuclear Export

**Figure 7:**
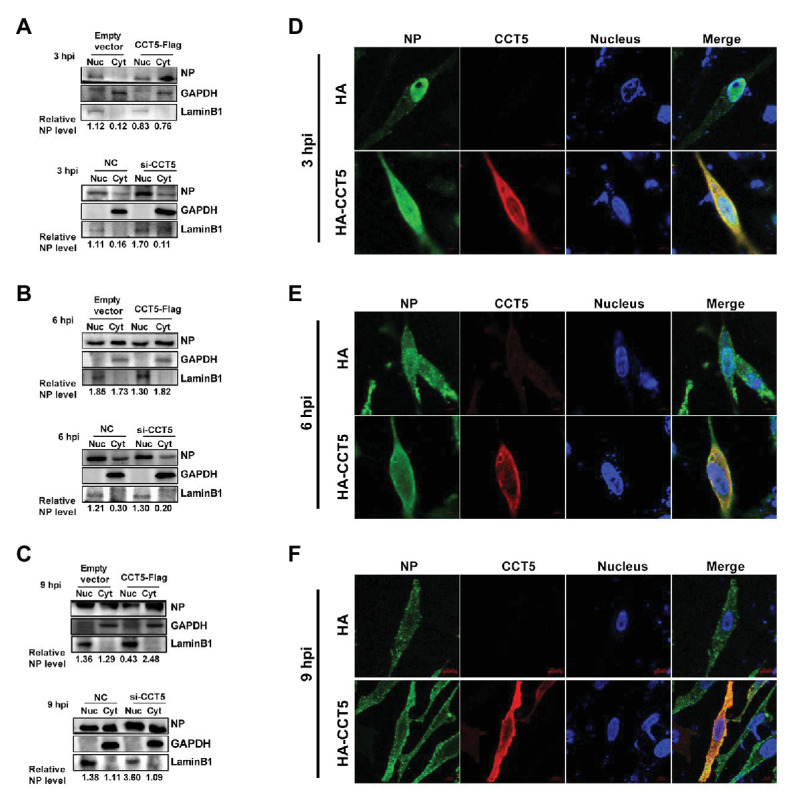
Effect of CCT5 on nucleoprotein (NP) distribution in DF-1 cells. **(A–C)** DF-1 cells were transfected with siCCT5#1 or CCT5-Flag plasmid for 24 h and then infected with 0.1 MOI H5N6-GFP. At 3, 6, and 9 h postinfection, cytoplasm, and nucleoplasm were separated for Western blot analysis. LaminB1 was used as a nuclear loading control. GAPDH was used as a cytosolic loading control. **(D–F)** The DF-1 cells were treated following the same procedure as above and then subjected to stain with CCT5 or NP primary antibody followed by secondary antibodies as described in Materials and Methods section. The localization of CCT5 and viral NP was observed under confocal microscopy.

Our data demonstrated that CCT5 interacted with NP protein and promoted the replication of the influenza virus. Because vRNP nuclear import or export is crucial for completing the influenza virus’ life cycle, we were interested in testing the effect of CCT5 on the distribution of NP in DF-1 cells. Thus, the DF-1 cells were transfected with HA-CCT5 expressing vector or CCT5 siRNA followed by infection with 0.1 MOI of H5N6 virus. Then, nuclear and cytoplasmic fractionation was performed at 3, 6, and 9 h after infection, respectively. The results showed that ectopic overexpression of CCT5 reduced the nuclear NP amount yet increased the cytoplasm NP amount. Reversely, knockdown of CCT5 expression resulted in an opposite effect on NP distribution (Figures 7A–C). Consistently, the data of immunofluorescence suggested the retention of NP protein in the cytoplasm in CCT5 overexpressed cells (Figures 7D–F). Taken together, CCT5 might play an essential role in vRNP nuclear export.

## Discussion

The vRNP complex is indispensable for the influenza virus to initiate and complete the transcription and replication of the viral genome in the nucleus of infected cells (Mayer et al., 2007; Tafforeau et al., 2011). Importantly, the vRNP complex is encapsidated by the viral NP protein, which contains at least two nuclear localization signals that are responsible for vRNP import into the nucleus through cellular importin alpha/beta pathway (Gabriel et al., 2008; Resa-Infante et al., 2008; Zhong et al., 2012). To date, many host proteins are unveiled in human cells to regulate the vRNP complex. However, the interplay between IAV and the avian host is less understood. In this study, in avian DF-1 cells, we identified CCT5 as a novel interacting partner of influenza NP protein through IP/MS. Our results showed that NP and CCT5 could colocalize and interact with each other in both overexpression and normal infection conditions. Moreover, the CCT5 expression level was largely promoted by an influenza virus infection, which, in turn, facilitated viral replication.

Influenza A Virus must utilize host various machinery to support its life cycle in infected cells. As an important part of viral components, the vRNP complex is an important determinant of virus pathogenicity and host adaptation, whose function can be affected by host factors (Cros et al., 2005; Ozawa et al., 2007; Zhang et al., 2016). For example, a recent report uncovered that host PLSCR1 could form a trimeric complex with NP and importin α to block the incorporation of importin β, thereby impairing the nuclear import of NP and suppressing virus replication (Luo et al., 2018). To identify proteins that potentially interact with NP in DF-1 cells, we employed affinity purification and mass spectrometry. We identified 72 cellular proteins that copurified with the reconstituted NP. Of these identified proteins, HSP70 has been shown to interact with vRNP complex and inhibit its assembly in host cells (Conti et al., 2001; Hirayama et al., 2004). Besides, some proteins like NOP56, FARSA, CCT5, and RUVBL1 were also indicated to associate with vRNP in a previous report (Heaton et al., 2016). Our follow-up results showed that silencing NOP56 and RUVBL1 did not affect influenza vRNA levels in DF-1 cells. In contrast, impaired expression of FARSA, CCT5, and MTHFD1L reduced viral mRNA abundance, indicating their potential regulatory role on virus replication.

Chaperonin containing TCP1 is a group II chaperonin complex for its role in the folding of newly synthesized polypeptides (Freund et al., 2014). Previously, in human A549 cells, Fislova et al. (2010) determined that CCT5 could interact with influenza PB2 but not with PB1 and PA; however, whether it interacted with NP had not been investigated. In the present study, we provided evidence that avian CCT5 could interact with influenza PB2 and PB1 but not with PA. More importantly, both co-IP and immunofluorescence data indicated that avian CCT5 had a favorable association with influenza NP protein under overexpression or viral infection. Our data, together with the previous finding of others, suggested that CCT5 most likely formed a multicomponent complex with vRNP to control the viral life cycle in both human and avian cells, although the involved mechanism might vary across different species.

We showed that the overexpressed CCT5 largely inhibited the nuclear import of the NP. However, it was promoted by knockdown of CCT5. Notably, our data also demonstrated that the enhanced NP nuclear import facilitated the replication of the influenza virus, although the mechanism for how CCT5 promoted this process was absent in this study. Previous reports had documented that the chaperone-assisted protein-folding pathway played a critical role in influenza replication (Guan et al., 2010; Jirakanwisal et al., 2015). By acting as a chaperone molecule, it is possible that CCT5 most likely regulates the transportation of NP or vRNP between nuclear and cytoplasm and, in turn, favors viral replication. Interestingly, we found that the ectopic expression of CCT5 enhanced viral polymerase activity (Supplementary Figure 3), which potentially caused a prominent positive effect on virus production.

In summary, here, we reported that avian CCT5, whose expression could be upregulated by IAV infection, is an interacting partner of the viral NP protein. The association of CCT5 with NP appears to enhance virus replication seeing that overexpression of CCT5 increased the synthesis of vRNA, cRNA, and mRNA and the virus titer in cell cultures. In contrast, the knockdown of CCT5 had the opposite effect. Importantly, mechanical studies revealed that CCT5 promoted the nuclear export of NP and increased the viral polymerase activity during virus infection. Overall, our data suggest that CCT5 plays an important role in supporting influenza virus replication, which may hold the potential as an anti-influenza target.

## Data Availability Statement

All datasets generated for this study are included in the article/Supplementary Material.

## Author Contributions

XZ, LZ, and MJ conceived and designed the experiments. XZ and CQ performed the experiments. XL and LZ analyzed the data. KH and XS contributed to the reagents and materials. LZ and MJ wrote the paper. All authors contributed to the article and approved the submitted version.

### Conflict of Interest

The authors declare that the research was conducted in the absence of any commercial or financial relationships that could be construed as a potential conflict of interest.
