# Supplementary material for: Avian Chaperonin Containing TCP1 Subunit 5 Supports Influenza A Virus Replication by Interacting With Viral Nucleoprotein, PB1, and PB2 Proteins
Source: Front Microbiol. 2020 Oct 15;11:538355. doi: 10.3389/fmicb.2020.538355 (PMC7593399; doi:10.3389/fmicb.2020.538355)

Supplementary Material

# Supplementary Data

## Small interfering RNAs used in this study (5' to 3')

siFARSA #1

GCUGCUAGAAGUCACUCUUTT AAGAGUGACUUCUAGCAGCTT

siFARSA #2

GGAACUUUGAUGCCCUCUUTT AAGAGGGCAUCAAAGUUCCTT

siFARSA #3

GGAGGUCUUCAGCUACCAUTT AUGGUAGCUGAAGACCUCCTT

siMTHFD1L #1

GCUAUUAUCCAGGCACAUATT UAUGUGCCUGGAUAAUAGCTT

siMTHFD1L #2

CCACCAGUCUGAAGGAUAUTT AUAUCCUUCAGACUGGUGGTT

siMTHFD1L #3

CCAAAUUACUCAGCUCUUUTT AAAGAGCUGAGUAAUUUGGTT

siNOP56 #1

GGGCCAAGGUGAAGUUCAATT UUGAACUUCACCUUGGCCCTT

siNOP56 #2

CCCUGUUCAGGGCUUUGAATT UUCAAAGCCCUGAACAGGGTT

siNOP56 #3

GCAAGAACCUGGAGGUCAUTT AUGACCUCCAGGUUCUUGCTT

siRUVBL1 #1

GCACUGGAAUCUUCUAUUUTT AAAUAGAAGAUUCCAGUGCTT

siRUVBL1 #2

GCUGAAGAGUAUGUUCCUUTT AAGGAACAUACUCUUCAGCTT

siRUVBL1 #3

GGAUGUAGUGUCUCCUCAUTT AUGAGGAGACACUACAUCCTT

siCCT5 #1

GCAAGUACUCUGAGAACAUTT AUGUUCUCAGAGUACUUGCTT

siCCT5 #2

GCACAACGAAGGACAGAAUTT AUUCUGUCCUUCGUUGUGCTT

siCCT5 #3

GCAGAUGCCUUGGAGGUAATT UUACCUCCAAGGCAUCUGCTT

## PCR Primers for vector construction (5' to 3')

CCT5-Flag (Kpn1)-F AAA*GGTACC*ATGTCGGCCATGGGGACGCTGG

CCT5-Flag (BamH1)-R TTT*GGATCC*CTCTTCAGATTCTCCAGGCCTA

CCT5-HA (EcoR I) -F AAA*GAATTC*ATGTCGGCCATGGGGACGCTGG

CCT5-HA (Xho I)-R TTT*CTCGAG*CTCTTCAGATTCTCCAGGCCTA

NP-HA (EcoR I)-F AAA*GAATTC*ATGGCGTCTCAAGGCACCAAACG

NP-HA (Xho I)-R TTT*CTCGAG*TTATTTTTCTGCACTACGCAGGG

PB2-HA (Sac I) -F AAA*GAGCTC*ATGAACAGAATAAAGGAACTAAG

PB2-HA (Xho I)-R TTT*CTCGAG*TTATTTTTCTGCACTACGCAGGG

## RT-qPCR primers for chicken and influenza virus genes (5' to 3')

chRUVBL-F CATGAAGATCGAGGAGGTGAAG

chRUVBL-R CAGCCATCTTCTTGCTTTTGAT

chCCT5-F GGCTCTCAAGTCTCACATCAT

chCCT5-R ACCATCATCTTATCCAAGCCAT

chFARSA-F AGGTGATCGAGGCAGAGA

chFARSA-R CTTCATGGCGTCGCTTTG

chMTHFD1L-F AAATCTTTGGCCTCTCTGAAGA

chMTHFD1L-R CATTTCTTGAACCAAAGGAGCA

chNOP56-F TCATCAACATCGAGAGCTTCTC

chNOP56-R GATGAAGGTGGAGTGGAAGATG

chACTIN-RT-F AAATTGTGCGTGACATCAAGGA

chACTIN-RT-R AGGCAGCTGTGGCCATCTC

H5N1-DW-NP-F AACGACCGGAATTTCTGGAGAGG

H5N1-DW-NP-R CCGTACACACAAGCAGGCAAGC

H5N1-HM-NP-F AGTGGCCAGTGGATATGACTTT

H5N1-HM-NP-R TGCCATCCACACTAATTGACTC

H5N6-NP-F AACGACCGGAATTTCTGGAGAGG

H5N6-NP-R CCGTACACACAAGCAGGCAAGC

H5N6-PB2-F CAAGGAGACGTGGTGTTGGTAAT

H5N6-PB2-R CTAATTGATGGCCATCCGAATT

H5N6-PA-F CTATATGAAGCAATTGAGGAG

H5N6-PA-R TGGACAGTATGGATAGCAAAT

## Reverse transcription primers (5' to 3')

oligo(dT)18 TTTTTTTTTTTTTTTTTT

Primer for viral cRNA CCTTGTTTCTACT

vRNA (uni12) AGCAAAAGCAGG

# Supplementary Figures and Tables

## Supplementary Figures


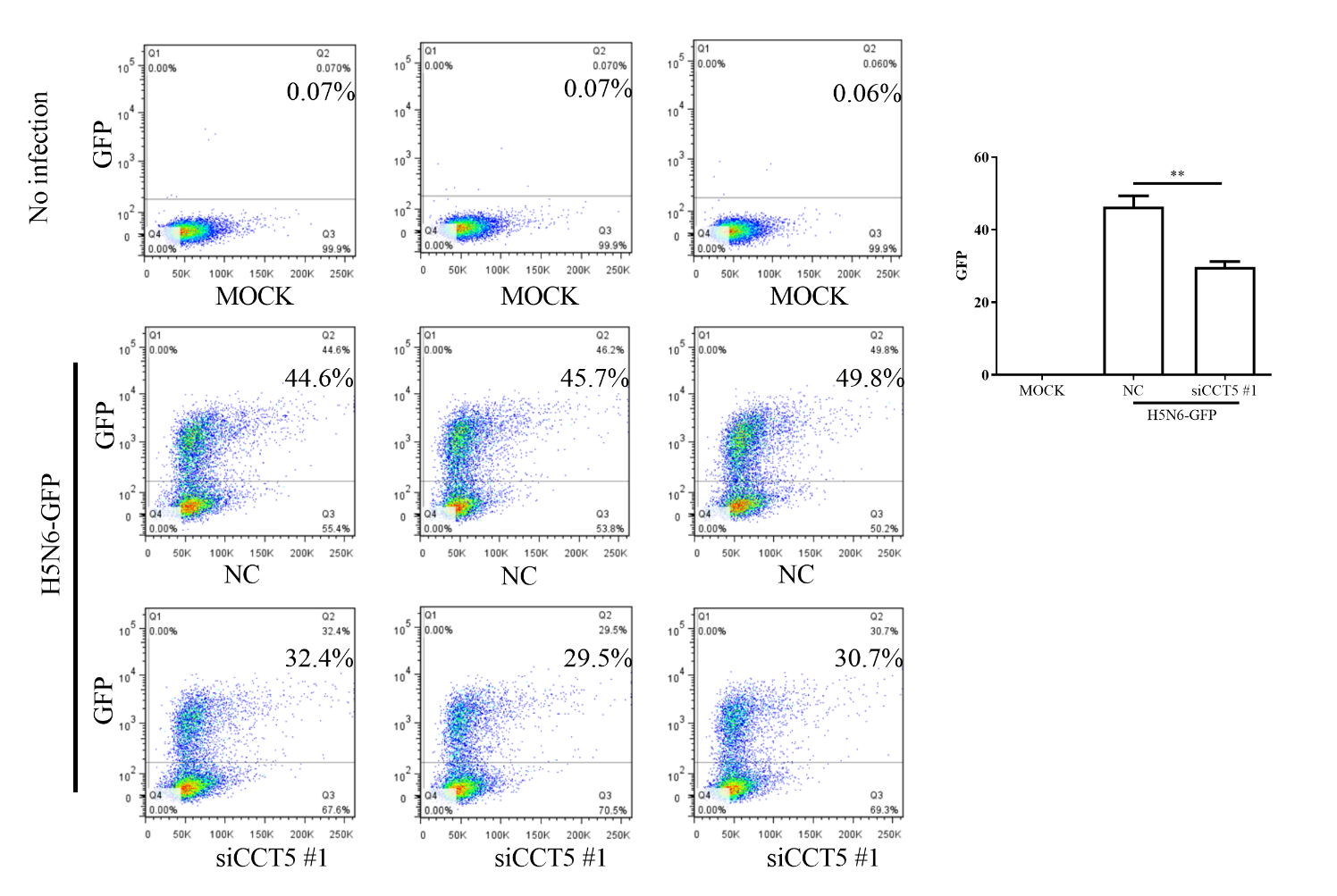


**Supplementary Figure 1.** The knockdown of CCT5 reduces H5N6-GFP replication by flow cytometry assay. DF-1 cells were transfected with siCCT5 #1 or NC for 24 h, followed by infection with 0.1 MOI of the H5N6-GFP virus for another 24 h. Then, the GFP ratio of DF-1 cells was measured by flow cytometry. Uninfected DF1 cells were used as negative control (**, *P* < 0.01, by *t*-test).


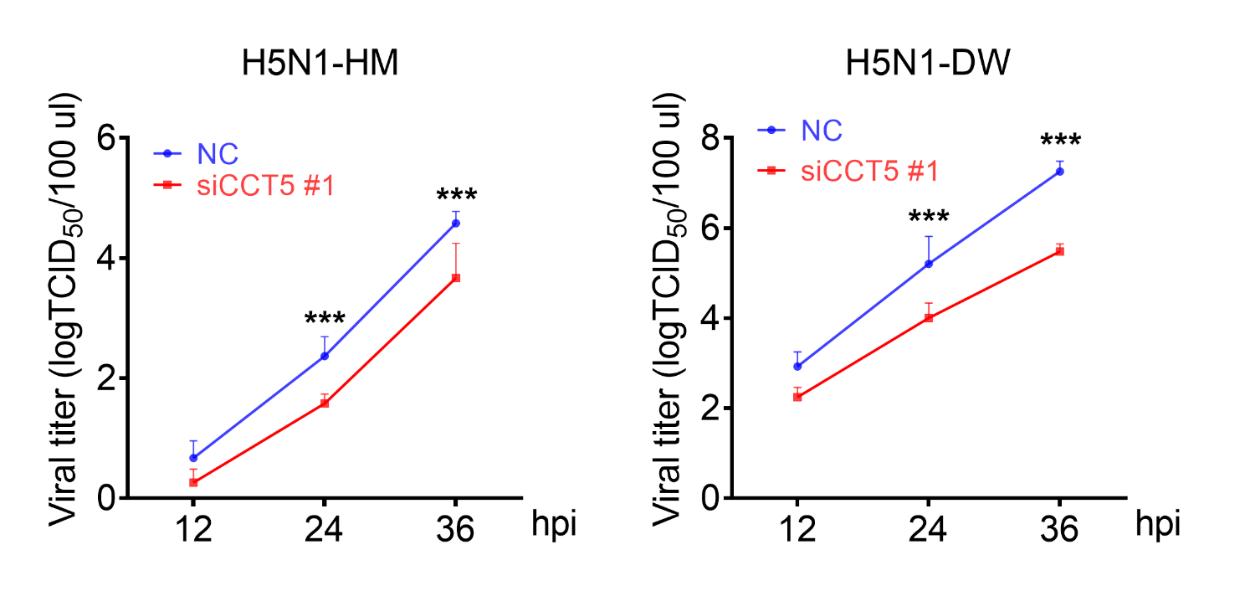


**Supplementary Figure 2.** The knockdown of CCT5 reduces the production of H5N1-HM and H5N1-DW viruses in DF-1 cells. DF-1 cells were transfected with siCCT5 #1 or NC for 24 h followed by infection with 0.1 MOI of H5N1-HM or H5N1-DW virus. Viral TCID_50_ in supernatants at each time point was determined. (***, *P* < 0.001, by *t*-test).


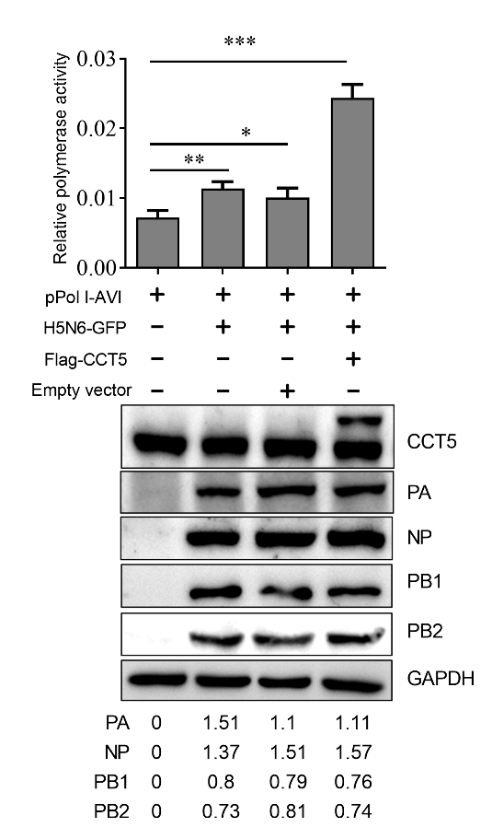


**Supplementary Figure 3.** Enhanced CCT5 expression promotes the polymerase activity of the influenza virus. The recombinant plasmids of viral polymerase, pPol I-AVI was co-transfected with CCT5 into DF1 cells followed by infection with 0.1 MOI H5N6-WT virus. The luciferase activity was determined at 12 h post-infection. The protein levels of CCT5, PB1, PB2, PA, and NP were determined by western blotting. (*, *P* < 0.05; **, *P* < 0.01; ***, *P* < 0.001, by *t*-test.).

## Supplementary Tables

Table 1. Seventy-two host proteins were identified by LC-MS/MS mass spectrometry.

| Accession | Description | Σ# Proteins | Σ# Unique Peptides | Σ# Peptides | Σ# PSMs | Score | # AAs | MW [kDa] | calc. pI |
| --- | --- | --- | --- | --- | --- | --- | --- | --- | --- |
| A0A1D5NW68 | ALB | 2 | 1 | 1 | 1 | 23.66 | 615 | 69.8 | 5.74 |
| A0A1D5P4L7 | TF | 6 | 4 | 4 | 5 | 64.72 | 705 | 77.8 | 7.12 |
| A0A1D5P9N5 | VTG1 | 3 | 11 | 11 | 20 | 458.78 | 1905 | 209.8 | 8.98 |
| A0A1D5PC92 | HIST1H2B5L | 6 | 1 | 1 | 2 | 32.75 | 126 | 13.9 | 10.24 |
| A0A1D5PV37 | VTG2 | 3 | 1 | 15 | 22 | 420.52 | 1109 | 124.6 | 9.10 |
| A0A1D5PVQ6 | LOC768978 | 3 | 1 | 1 | 5 | 75.64 | 442 | 50.4 | 5.60 |
| A0A1L1RMM1 | HBZ | 8 | 1 | 1 | 2 | 52.58 | 76 | 8.5 | 7.39 |
| A0A1L1RQL5 | SLC25A6 | 8 | 1 | 1 | 1 | 48.52 | 245 | 27.1 | 9.66 |
| A0A1L1RX27 | VIM | 6 | 2 | 2 | 2 | 39.52 | 313 | 36.8 | 4.84 |
| A0A2H4Y7R6 | OVA | 293 | 2 | 2 | 2 | 77.90 | 386 | 42.8 | 5.21 |
| B3VHV2 | HSP70 | 10 | 2 | 2 | 3 | 46.06 | 634 | 69.8 | 5.77 |
| B8YJN9 | LYZ | 4 | 1 | 1 | 1 | 64.77 | 147 | 16.2 | 9.10 |
| F1NDN6 | KRT12 | 4 | 1 | 3 | 7 | 202.06 | 509 | 53.7 | 5.01 |
| F1NFM8 | RSBN1 | 2 | 1 | 1 | 1 | 23.63 | 640 | 72.0 | 6.99 |
| F1NHI3 | DHX15 | 2 | 1 | 1 | 1 | 27.35 | 762 | 87.6 | 7.33 |
| P60706 | ACTB | 48 | 6 | 6 | 13 | 55.91 | 375 | 41.7 | 5.48 |
| A0A1D5PDV6 | RPS19 | 1 | 2 | 2 | 2 | 13.04 | 138.0 | 15.37 | 10.3 |
| Q5F411 | CCT5 | 1 | 1 | 1 | 1 | 22.59 | 541 | 59.7 | 5.74 |
| Q5ZJQ2 | FARSA | 1 | 1 | 1 |  | 3.16 | 443 | 49.5 | 8.92 |
| F1N8Z4 | RUVBL | 1 | 4 | 4 |  | 11.84 | 456 | 50.1 | 6.42 |
| F1NYI3 | RTCB | 1 | 3 | 3 |  | 4.55 | 505 | 55.2 | 7.24 |
| A0A1D5P629 | NOP56 | 3 | 7 | 7 | 11 | 57.90 | 535 | 59.8 | 9.10 |
| F1N8D4 | FLNB | 3 | 13 | 13 | 13 | 36.85 | 2555 | 273.8 | 5.67 |
| A0A1D5P833 | GSTM2 | 3 | 8 | 9 | 17 | 32.19 | 220 | 25.9 | 7.24 |
| F1NW23 | CLTC | 8 | 11 | 11 | 11 | 26.90 | 1675 | 191.5 | 5.74 |
| A0A1D5PS72 | EEF1A1 | 18 | 6 | 6 | 7 | 22.69 | 352 | 38.8 | 9.13 |
| P17785 | ANXA2 | 2 | 8 | 8 | 9 | 20.43 | 339 | 38.6 | 7.33 |
| R9PXN7 | HPGDS | 3 | 7 | 7 | 9 | 19.56 | 199 | 22.7 | 7.28 |
| A0A1D5NT70 | GSTAL1 | 4 | 3 | 3 | 5 | 19.38 | 221 | 25.3 | 8.65 |
| F1N8Y3 | CBR1 | 1 | 5 | 5 | 5 | 18.46 | 276 | 30.3 | 8.05 |
| A0A1L1RLK7 | ACTB | 34 | 3 | 3 | 7 | 14.01 | 254 | 28.5 | 5.33 |
| A0A1D5PBI5 | DDB1 | 2 | 5 | 5 | 5 | 12.88 | 1139 | 126.6 | 5.19 |
| A0A1L1RXL9 | VIM | 6 | 4 | 4 | 4 | 12.73 | 392 | 46.0 | 4.84 |
| F1NYA9 | EEF1B2 | 3 | 3 | 4 | 6 | 12.33 | 224 | 24.7 | 4.78 |
| F1NEF2 | CCT8 | 8 | 4 | 4 | 4 | 9.66 | 548 | 59.4 | 5.60 |
| O93510 | GSN | 5 | 3 | 3 | 3 | 8.18 | 778 | 85.8 | 6.32 |
| A0A1D5NZ30 | NCL | 2 | 3 | 3 | 3 | 7.70 | 691 | 75.4 | 4.92 |
| P14105 | MYH9 | 6 | 5 | 5 | 5 | 7.44 | 1959 | 226.4 | 5.57 |
| A0A1D5NVL7 | TPM1 | 24 | 1 | 3 | 3 | 7.37 | 278.0 | 32.17 | 4.8 |
| A0A1D5P9P3 | ACTN1 | 12 | 4 | 4 | 4 | 7.27 | 885 | 102.4 | 5.55 |
| A0A1D5PRB1 | TPM4 | 24 | 1 | 3 | 3 | 7.26 | 271 | 31.3 | 4.72 |
| F1N9V4 | LANCL1 | 4 | 4 | 4 | 4 | 7.24 | 339 | 38.5 | 7.68 |
| D0EKR3 | PPIA | 2 | 3 | 3 | 3 | 7.12 | 165 | 17.8 | 8.09 |
| A0A1L1RY04 | ATP5F1B | 4 | 2 | 2 | 2 | 6.54 | 353 | 38.2 | 5.20 |
| A0A1L1RNH7 | KRT7 | 11 | 1 | 2 | 2 | 6.04 | 352 | 39.0 | 5.08 |
| A0A1L1RY35 | ATP5A1Z | 8 | 2 | 2 | 2 | 5.85 | 449 | 49.0 | 9.07 |
| A0A1L1RZP1 | RPS2 | 3 | 1 | 1 | 2 | 5.42 | 184 | 19.9 | 10.13 |
| A0A1L1RIW5 | KRT5 | 8 | 1 | 2 | 2 | 5.35 | 584 | 61.1 | 8.84 |
| A0A1D5NZW9 | PGK1 | 4 | 2 | 2 | 2 | 5.03 | 389 | 41.5 | 7.49 |
| F1NNP6 | GLRX3 | 2 | 2 | 2 | 2 | 4.46 | 328 | 36.6 | 5.60 |
| A0A1D5P9V0 | PKM | 3 | 2 | 2 | 2 | 4.42 | 523 | 57.3 | 8.27 |
| A0A1D5NXH8 | RPS3A | 3 | 1 | 1 | 1 | 4.28 | 227 | 25.8 | 9.41 |
| A0A1D5PK69 | RPLP0 | 2 | 2 | 2 | 2 | 3.86 | 269 | 29.2 | 5.54 |
| P13648 | LMNA | 1 | 2 | 2 | 2 | 3.66 | 657 | 73.1 | 6.95 |
| G1K303 | HAGH | 3 | 1 | 1 | 1 | 3.38 | 260 | 29.0 | 6.90 |
| Q5ZIK9 | COPE | 1 | 1 | 1 | 1 | 3.36 | 308 | 34.3 | 5.14 |
| A0A1D5PKL0 | TERAL2 | 7 | 1 | 1 | 1 | 2.94 | 258 | 28.2 | 5.26 |
| A0A1L1RJY5 | HSPA8 | 6 | 1 | 1 | 1 | 2.92 | 198 | 21.7 | 4.94 |
| Q8JG64 | PDIA3 | 1 | 1 | 1 | 1 | 2.74 | 505 | 56.1 | 6.02 |
| E1BUN2 | EXOC4 | 1 | 1 | 1 | 1 | 2.72 | 976 | 111.0 | 6.55 |
| A0A1L1RW15 | GAPDH | 6 | 1 | 1 | 1 | 2.66 | 105 | 11.5 | 5.45 |
| A0A1D5PNR4 | PRDX3 | 4 | 1 | 1 | 1 | 2.65 | 101.0 | 11.21 | 5.8 |
| P18302-3 | DBN1 | 5 | 1 | 1 | 1 | 2.61 | 553 | 61.1 | 4.39 |
| A0A1D5PMQ5 | KRTC42L | 3 | 1 | 1 | 1 | 2.60 | 499 | 54.0 | 4.98 |
| E1C040 | HECTD1 | 2 | 1 | 1 | 1 | 2.59 | 2567 | 284.5 | 5.30 |
| Q5ZME1 | HNRNPA2B1 | 1 | 1 | 1 | 1 | 2.47 | 349 | 37.0 | 8.65 |
| Q90593 | HSPA5 | 1 | 1 | 1 | 1 | 2.46 | 652 | 72.0 | 5.22 |
| F1NZ86 | HSPA9 | 2 | 2 | 2 | 2 | 2.35 | 675 | 73.1 | 6.43 |
| A0A1D5P1F0 | NPM1 | 4 | 1 | 1 | 1 | 2.34 | 235 | 25.9 | 4.53 |
| Q5ZM58 | ACTR1A | 2 | 1 | 1 | 1 | 2.33 | 376 | 42.6 | 6.64 |
| E1C2P9 | KTN1 | 3 | 1 | 1 | 1 | 2.25 | 1364 | 155.9 | 5.86 |

Table 2. Candidate proteins overlapped with the study of Heaton et al.


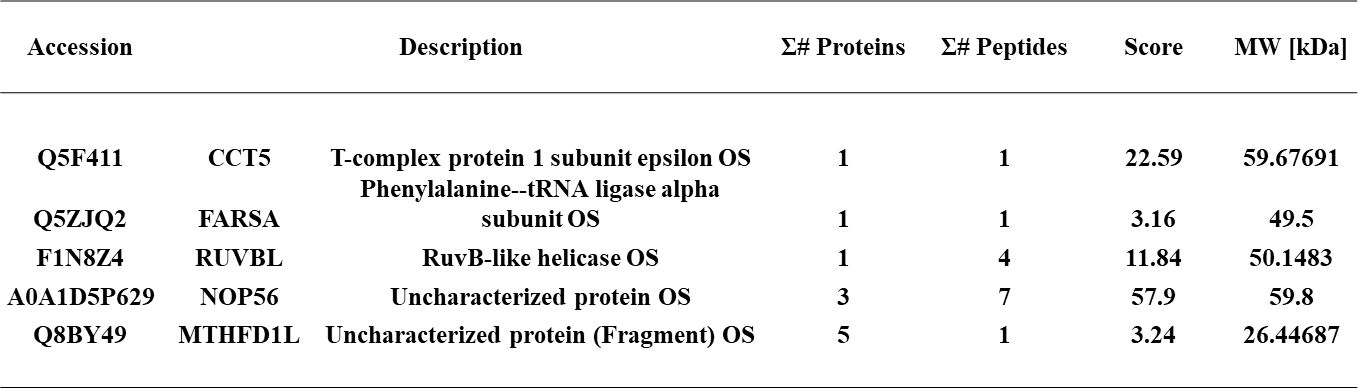

Supplement: Supplementary file 1 [file Data_Sheet_1.docx]
